# Supplementary material for: Amino acid residue at position 188 determines the UV-sensitive bistable property of vertebrate non-visual opsin Opn5
Source: Commun Biol. 2022 Jan 18;5:63. doi: 10.1038/s42003-022-03010-x (PMC8766551; doi:10.1038/s42003-022-03010-x)
Supplement: Supplementary file 2 — Supplementary Information [file 42003_2022_3010_MOESM2_ESM.pdf]

Supplementary information for

Amino acid residue at position 188 determines the UV-sensitive bistable property of vertebrate non-visual opsin Opn5

Authors: Chihiro Fujiyabu [1], Keita Sato [2], Yukimi Nishio [1], Yasushi Imamoto [1], Hideyo Ohuchi [2], Yoshinori Shichida [1,3], Takahiro Yamashita [1*]

Affiliations: [1] Department of Biophysics, Graduate School of Science, Kyoto University, Kyoto 606-8502, Japan; [2] Department of Cytology and Histology, Okayama University Graduate School of Medicine, Dentistry and Pharmaceutical Sciences, Okayama 700-8558, Japan; [3] Research Organization for Science and Technology, Ritsumeikan University, Shiga 525-8577, Japan

*Corresponding Author: Takahiro Yamashita, Department of Biophysics, Graduate School of Science, Kyoto University, Kyoto 606-8502, Japan.
Email: yamashita.takahiro.4z@kyoto-u.ac.jp

Supplementary Figure 1

```
                             10         20         30         40         50
                     ....|....| ....|....| ....|....| ....|....| ....|....|
Bovine Rhodopsin     MNGTEGPNFY VPFSNKTGVV RSPFEAPQYY LAEPWQFSML AAYMFLLIML
Squid Rhodopsin      --MGRDLRDN ETWWYNPSIV VHPHWR-EFD QVPDAVYYSL GIFIGICGII
Mouse Opn5m          MALNHTALPQ DERLPHYLRD EDPFAS-KLS WEADLVAGFY LTIIGILSTF
Xenopus Opn5m        MAGNSSYREE SGYIPHYERD SDPFAS-KLS READIFAGVY LMAIGILSTL
Chicken Opn5L1       ---------- ----MDPSFG NSTFQS-KIT EAADIVVGTC YMVFGICSLC
Xenopus Opn5L1a      ---------- ----MGLTK- NTSFHS-NIP HTADNIFGII YILFGLCSVL

                             60         70         80         90        100
                     ....|....| ....|....| ....|....| ....|....| ....|....|
Bovine Rhodopsin     GFPINFLTLY VTVQHKKLRT PLNYILLNLA VADLFMVFG- GFTTTLYTSL
Squid Rhodopsin      GCGGNGIVIY LFTKTKSLQT PANMFIINLA FSDFTFSLVN GFPLMTISCF
Mouse Opn5m          G---NGYVLY MSSRRKKKLR PAEIMTINLA VCDLGISVV- GKPFTIISCF
Xenopus Opn5m        G---NGYVIY MACSRKKKLR PAEIMTINLA VCDLGISVT- GKPFAIVSCF
Chicken Opn5L1       G---NSILLY ISYKKKHLLK PAEYFIINLA ISDLAMTLT- LYPLAVTSSL
Xenopus Opn5L1a      G---NSTLLY ISYKRRHLLK PAEYFIVNLA LSDLAMTVT- LYPLAITSSF

                            110        120        130        140        150
                     ....|....| ....|....| ....|....| ....|....| ....|....|
Bovine Rhodopsin     HGYFVFGPTG CNLEGFFATL GGEIALWSLV VLAIERYVVV CKPMSN-FRF
Squid Rhodopsin      LKKWIFGFAA CKVYGFIGGI FGFMSIMTMA MISIDRYNVI GRPMAASKKM
Mouse Opn5m          CHRWVFGWFG CRWYGWAGFF FGCGSLITMT AVSLDRYLKI CYLSYG-VWL
Xenopus Opn5m        SHRWVFGWNA CRWYGWAGFF FGCGSLITLT VVSLDRYLKI CHLRYG-TWL
Chicken Opn5L1       SHRWLYGKHI CLFYAFCGLF FGICSLSTLT LLSVVCCLKI CFPAYG-NRF
Xenopus Opn5L1a      SHRWLYGRHV CLFYAFCGVL FGICSLSTVT LLSTICCMKV CFPVYG-NRF

                            160        170        180        190        200
                     ....|....| ....|....| ....|....| ....|....| ....|....|
Bovine Rhodopsin     GENHAIMGVA FTWVMALACA APPLVGWSRY IPEGMQCSCG IDYYTPHEET
Squid Rhodopsin      SHRRAFIMII FVWLWSVLWA IGPIFGWGAY TLEGVLCNCS FDYISRDSTT
Mouse Opn5m          KRKHAYICLA VIWAYASFWT TMPLVGLGDY APEPFGTSCT LDWWLAQASG
Xenopus Opn5m        KRRHAFIALA VIWAYATLWA TLPLVGVGNY APEPFGTTCT LDWWLAQASV
Chicken Opn5L1       RRKHGQILIA CAWTYAAIFA CSPLAHWGEY GEEPYGTACC IDWQSTNVDV
Xenopus Opn5L1a      GHKQGCFLVA CAWLYAAIFA FSPLLHWGEY GAEPYGTACC IDWYSSNKSR

                            210        220        230        240        250
                     ....|....| ....|....| ....|....| ....|....| ....|....|
Bovine Rhodopsin     NNESFVIYMF VVHFIIPLIV IFFCYGQLVF TVK------- -----EAAAQ
Squid Rhodopsin      R--SNILCMF ILGFFGPILI IFFCYFNIVM SVSNHEKEMA AMAKRLNAKE
Mouse Opn5m          GGQVFILSIL FFCLLLPTAV IVFSYAKIIA KVK------- SSSKEVAHFD
Xenopus Opn5m        KGQIFVLSML FFCLLFPTMV IVFSYAKIIA KVK------- SSAKEVAHFD
Chicken Opn5L1       MSMSYTVVLF VLCFILPCGV IVTSYSLILV TVK------- ESRKAVEQH-
Xenopus Opn5L1a      VAMSYTTTLF VLCFVIPCGI IITSYTLILV TVK------- DSRKAVEQHG

                            260        270        280        290        300
                     ....|....| ....|....| ....|....| ....|....| ....|....|
Bovine Rhodopsin     QQESATTQKA EKEVTRMVII MVIAFLICWL PYAGVAFYIF THQGSDFGPI
Squid Rhodopsin      LRKAQAGANA EMRLAKISIV IVSQFLLSWS PYAVVALLAQ FGPLEWVTPY
Mouse Opn5m          SRIHS-SHVL EVKLTKVAML ICAGFLIAWI PYAVVSVWSA FGRPDSIPIQ
Xenopus Opn5m        TRNQN-NHTL EIKLTKVAML ICAGFLIAWF PYAVVSVWSA FGQPDSIPIE
Chicken Opn5L1       VSGPTRINNV QTITAKLSIA VCIGFFAAWS PYAIIAMWAA FGSIDKIPPL
Xenopus Opn5L1a      VAGPSSMNNV QIIIVKLSIA VCIGFFTAWS PYAVIAMWAA FGSIDIIPPL

                            310        320        330        340        350
                     ....|....| ....|....| ....|....| ....|....| ....|....|
Bovine Rhodopsin     FMTIPAFFAK TSAVYNPVIY IMMNKQFRN- ---------- -CMVTTLCCG
Squid Rhodopsin      AAQLPVMFAK ASAIHNPMIY SVSHPKFREA ISQTFP---- WVLTCCQFDD
Mouse Opn5m          LSVVPTLLAK SAAMYNPIIY QVIDYRF--- ---------- ---ACCQAG-
Xenopus Opn5m        LSVVPTMMAK SASMYNPIIY QVIDCKP--- ---------- ---ACCKKDK
Chicken Opn5L1       AFAIPAVFAK SSTLYNPIIH LLLKPNFRSN IAKDFTVIQQ LCVRCCFCVK
Xenopus Opn5L1a      VFAVPAVFAK SSTIYNPIIY LFLKPNFRNI LAKYFPAAQE ICTRSCLYMD

                            360        370        380        390        400
                     ....|....| ....|....| ....|....| ....|....| ....|....|
Bovine Rhodopsin     KNPLGDDEAS TTVSKTETSQ VAPA------ ---------- ----------
Squid Rhodopsin      KETEDDKDAE TEIPAGESSD AAPSADAAQM KEMMAMMQKM QQQQAAYPPQ
Mouse Opn5m          GLRGTKKKSL EDFRLHTVTA VRKSSAVLEI HPESSSRFTS ----------
Xenopus Opn5m        SLQNTTS--- ---RVYTIST FRKS------ --TTSAR--- ----------
Chicken Opn5L1       ELQT--YRST FNTGLRTFKG KNESSCNALP IMEGCSYFPS EKGSHTFECF
Xenopus Opn5L1a      SLNSCHYLPV IQLFHKLLNK RNTPASDSGK SMEEYPCYSC DQCKDTFEYF

                            410        420        430        440        450
                     ....|....| ....|....| ....|....| ....|....| ....|....|
Bovine Rhodopsin     ---------- ---------- ---------- ---------- ----------
Squid Rhodopsin      GYAPPPQGYP PQGYPPQGYP PQGYPPQGYP PPPQGAPPQG APPAAPPQGV
Mouse Opn5m          ---------- -----AHVMD GESHSNDGDC GKK------- ----------
Xenopus Opn5m        ---------- ---------- ---------- ---------- ----------
Chicken Opn5L1       KSYPNCFQER LSTMGCHLQD CESLENDLQV EVTQGSRNSM KVVEQEEKST
Xenopus Opn5L1a      KNYPHHCHEM LGPTPHTKQD GSCLDSNEQA MRKNSAKKSI KVIVHGQKTS

                            460        470
                     ....|....| ....|....| .
Bovine Rhodopsin     ---------- ---------- -
Squid Rhodopsin      DNQAYQA--- ---------- -
Mouse Opn5m          ---------- ---------- -
Xenopus Opn5m        ---------- ---------- -
Chicken Opn5L1       ELDNLEITLE AVPVSCTFTD L
Xenopus Opn5L1a      ESDDLEITLE VIPVCSKCVY -
```

Supplementary Fig. 1 Comparison of the amino acid sequences among bovine rhodopsin, squid rhodopsin, Opn5m and Opn5L1.

Amino acid sequences of bovine rhodopsin (K00506), squid (*Todarodes pacificus*) rhodopsin (X70498), mouse Opn5m (AY318865), *Xenopus tropicalis* Opn5m (XM_002935990), chicken Opn5L1 (AB368181) and *X. tropicalis* Opn5L1a (XM_031904599) were aligned using ClustalW 2.1 [1]. Three residues at positions 167, 188 and 212 are highlighted in red. The sequence similarity between squid rhodopsin and *X. tropicalis* is about 28 %.

Supplementary Figure 2

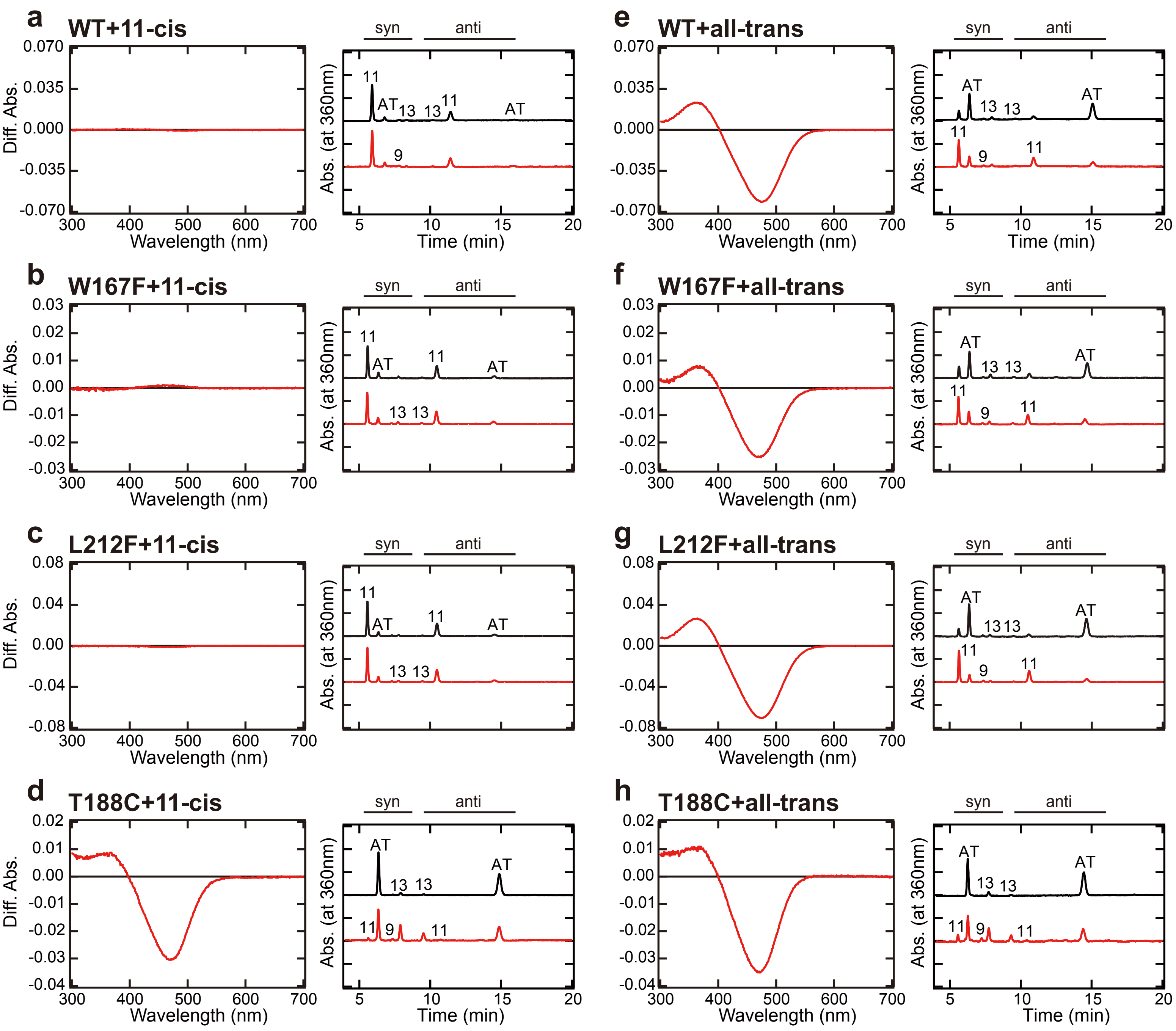

Supplementary Fig. 2 Spectral changes and retinal configuration changes of wild-type and W167F, L212F and T188C mutant Opn5m proteins

a-d (left) Spectral change caused by yellow light (>500 nm) irradiation of Opn5m wild-type (**a**) and W167F (**b**), L212F (**c**) and T188C (**d**) mutant proteins purified after the addition of 11-*cis* retinal to the collected cell membranes. Difference spectrum was calculated based on the spectra shown in Figs. 1b-1e. (right) Retinal isomers before irradiation (black) and after yellow light (>500 nm) irradiation (red) extracted from Opn5m wild-type (**a**) and W167F (**b**), L212F (**c**) and T188C (**d**) mutants purified after the addition of 11-*cis* retinal to the collected cell

membranes (Figs. 1b-1e). Retinal configurations were analyzed with HPLC after extraction of the chromophore as retinal oximes (*syn* and *anti* forms of 9-*cis*, 11-*cis*, 13-*cis*, and all-*trans* retinal oximes). **e-h** (left) Spectral change caused by yellow light (>500 nm) irradiation of Opn5m wild-type (**e**) and W167F (**f**), L212F (**g**) and T188C (**h**) mutant proteins purified after the addition of all-*trans* retinal to the collected cell membranes. Difference spectrum was calculated based on the spectra shown in Figs. 1b-1e. (right) Retinal isomers before irradiation (black) and after yellow light (>500 nm) irradiation (red) extracted from Opn5m wild-type (**e**) and W167F (**f**), L212F (**g**) and T188C (**h**) mutants purified after the addition of all-*trans* retinal to the collected cell membranes (Figs. 1b-1e).

Supplementary Figure 3

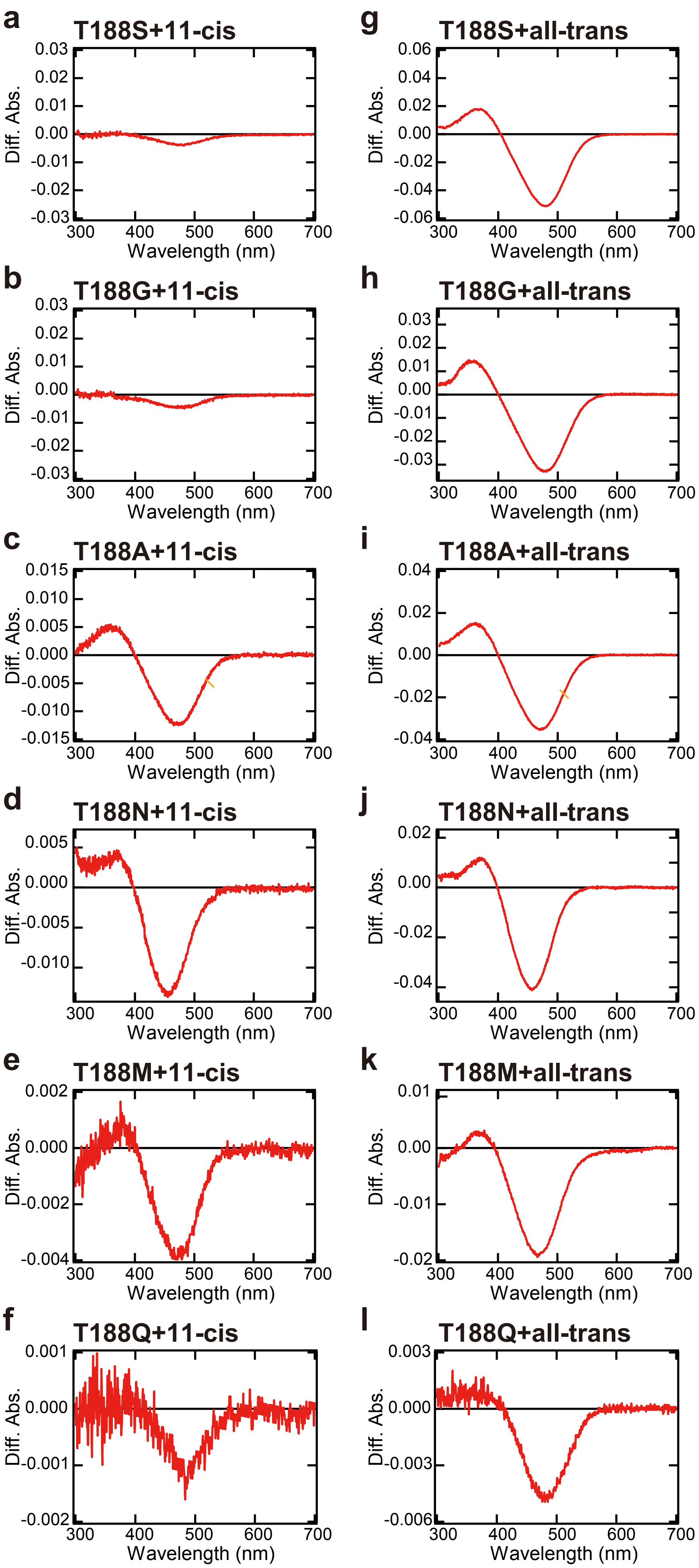

Supplementary Fig. 3 Spectral changes of Opn5m Thr188 mutant proteins after the addition of 11-*cis* or all-*trans* retinal

a-f Spectral change caused by yellow light (>500 nm) irradiation of Opn5m T188S (**a**), T188G (**b**), T188A (**c**), T188N (**d**), T188M (**e**), and T188Q (**f**) mutant proteins purified after the addition of 11-*cis* retinal to the collected cell membranes. Difference spectrum was calculated based on the spectra shown in Figs. 2a-2f. **g-l** Spectral change caused by yellow light (>500 nm) irradiation of Opn5m T188S (**g**), T188G (**h**), T188A (**i**), T188N (**j**), T188M (**k**), and T188Q (**l**) mutant proteins purified after the addition of all-*trans* retinal to the collected cell membranes. Difference spectrum was calculated based on the spectra shown in Figs. 2g-2l.

Supplementary Figure 4

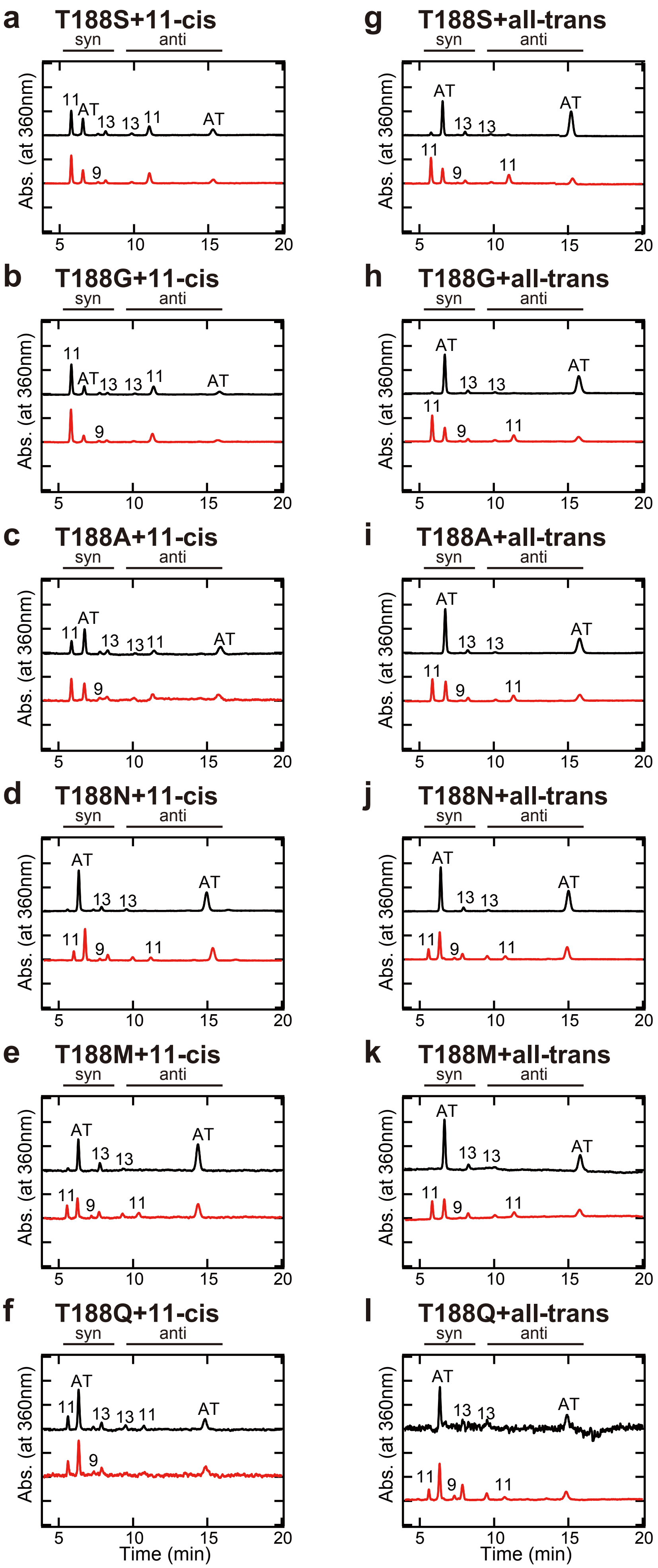

Supplementary Fig. 4 HPLC chromatograms of retinal isomer analysis

Retinal configurations were analyzed with HPLC after extraction of the chromophore as retinal oximes (*syn* and *anti* forms of 9-*cis*, 11-*cis*, 13-*cis*, and all-*trans* retinal oximes). **a-f** Retinal isomers before irradiation (black) and after yellow light (>500 nm) irradiation (red) were extracted from Opn5m T188S (**a**), T188G (**b**), T188A (**c**), T188N (**d**), T188M (**e**), and T188Q (**f**) mutant proteins purified after the addition of 11-*cis* retinal to the collected cell membranes (Figs. 2a-2f, right). **g-l** Retinal isomers before irradiation (black) and after yellow light (>500 nm) irradiation (red) were extracted from Opn5m T188S (**g**), T188G (**h**), T188A (**i**), T188N (**j**), T188M (**k**), and T188Q (**l**) mutant proteins purified after the addition of all-*trans* retinal to the collected cell membranes (Figs. 2g-2l, right). AT, all-*trans* retinal; 9, 9-*cis* retinal; 11, 11-*cis* retinal; 13, 13-*cis* retinal.

Supplementary Figure 5

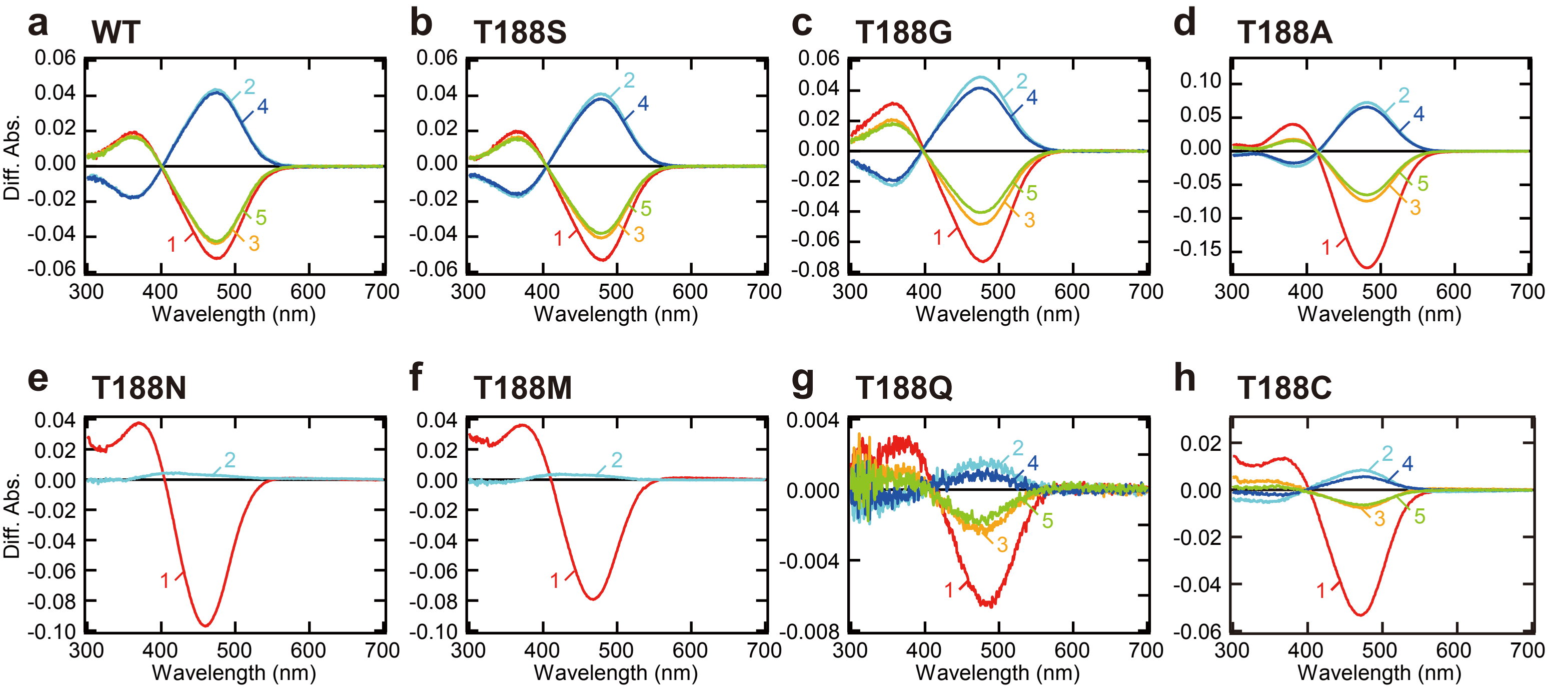

Supplementary Fig. 5 Spectral changes of Opn5m wild-type and Thr188 mutant proteins

Spectral changes of Opn5m wild-type (**a**) and T188S (**b**), T188G (**c**), T188A (**d**), T188N (**e**), T188M (**f**), T188Q (**g**) and T188C (**h**) mutant proteins caused by yellow light (>500 nm) irradiation (curve 1), subsequent UV light (360 nm) irradiation (curve 2), yellow light re-irradiation (curve 3), UV light re-irradiation (curve 4) and yellow light re-irradiation (curve 5) were calculated based on the spectra shown in Fig. 3.

Supplementary Figure 6

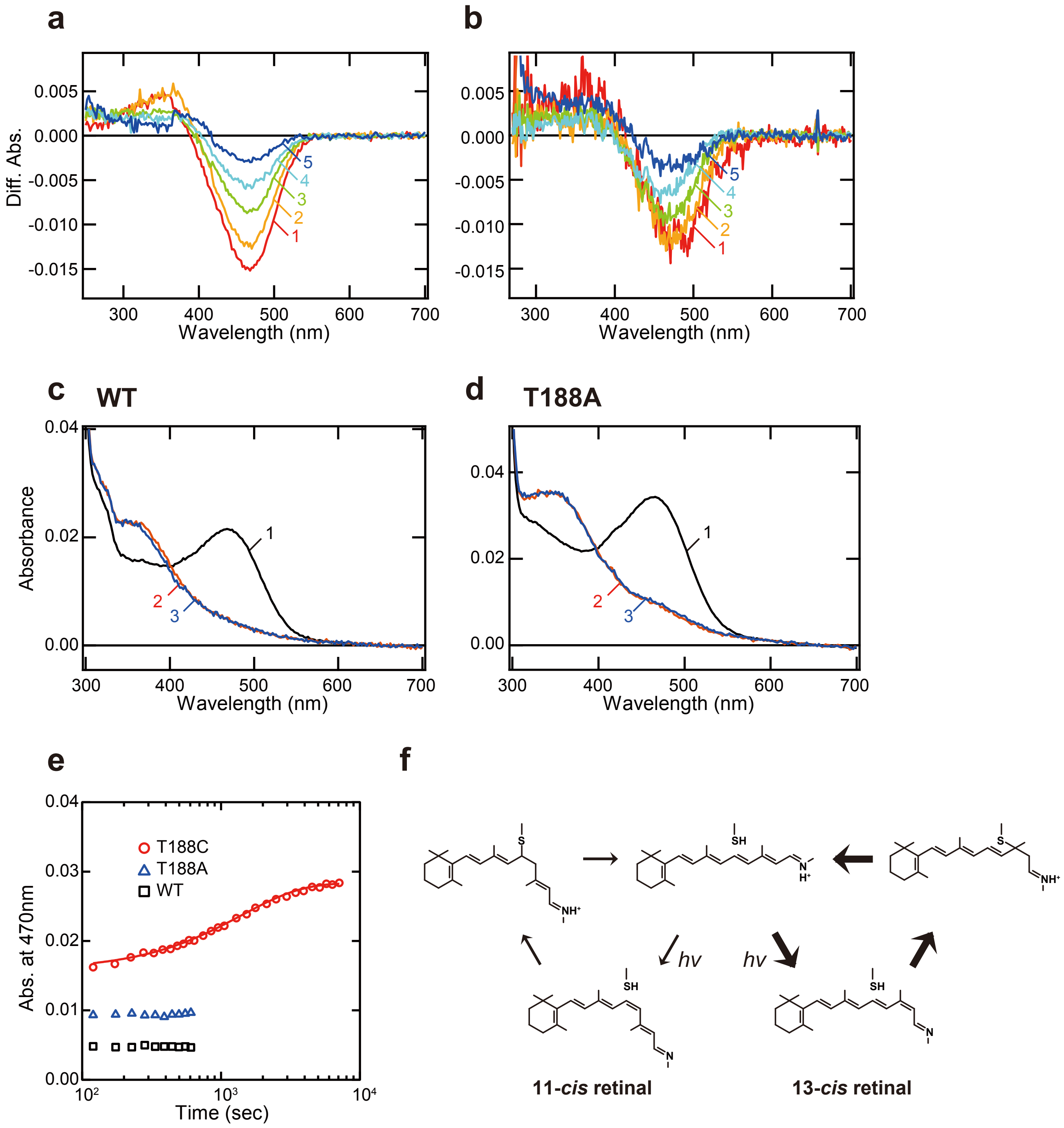

Supplementary Fig. 6 Spectral changes of T188C mutant Opn5m protein

a Spectral changes of Opn5m T188C mutant protein at 10 °C. Difference spectra were obtained by subtracting the spectrum before irradiation (curve 1 of Fig. 4a) from the spectra measured after yellow light (>500 nm) irradiation (curves 2-6 of Fig. 4a) (curves 1-5, respectively). **b** Spectral changes of Opn5m T188C mutant protein at 37 °C. Difference spectra were obtained by subtracting the spectrum before irradiation (curve 1 of Fig. 4b) from the spectra measured after yellow light (>500 nm) irradiation (curves 2-6 of Fig. 4b) (curves 1-5, respectively).

c,d Absorption spectra of Opn5m wild-type (**c**) and T188A mutant proteins (**d**) purified after the addition of all-*trans* retinal to the medium of the transfected cultured cells. The spectra were recorded in the dark (curve 1) and 0 min (curve 2) and 30 min (curve 3) after yellow light (>500 nm) irradiation at 10 °C. **e** Absorption change of Opn5m wild-type and T188C and T188A mutant proteins at 470 nm after light irradiation at 10 °C. Time course of the absorbance of T188C mutant protein at 470 nm was fitted with a single-exponential function (τ= 1405.2 sec). **f** Proposed model of retinal structural changes during the photocyclic reaction of Opn5m T188C mutant protein. Opn5m T188C protein binds all-*trans* retinal in the dark and photoisomerizes it into 13-*cis* and 11-*cis* retinals. These *cis* isomers are expected to form an adduct with the thiol group of Cys188. This accelerates the thermal isomerization of the retinal to all-*trans* form to recover to the original dark state.

Supplementary Figure 7

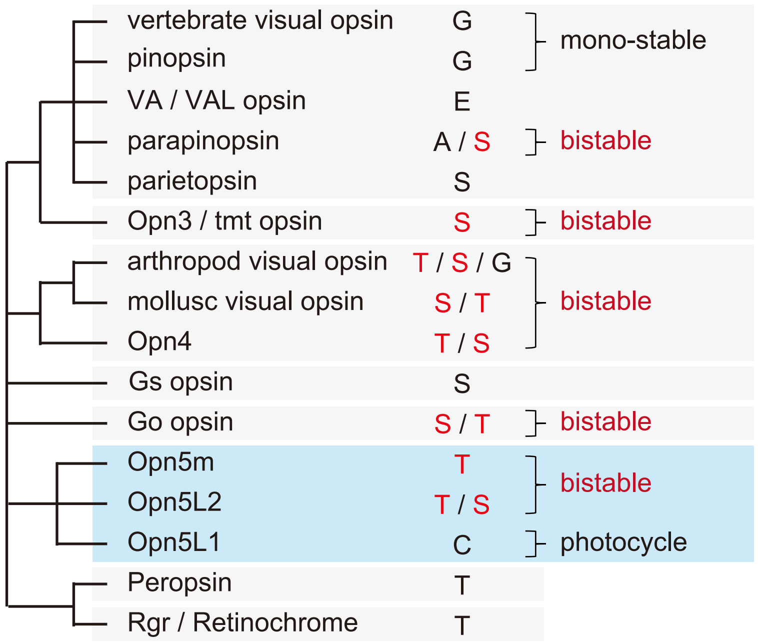

Supplementary Fig. 7 Phylogenic view of opsin family and amino acid residue at position 188 Most bistable opsins, including parapinopsin, Opn3/tmt opsin, Gq-coupled opsins, Go-coupled opsins and Opn5m/Opn5L2, have a threonine or serine residue at position 188. By contrast, Opn5L1 uniquely has a cysteine residue at position 188.

Supplementary References

1 Larkin, M. A. *et al*. Clustal W and Clustal X version 2.0. *Bioinformatics* **23**, 2947-2948, doi:10.1093/bioinformatics/btm404 (2007).
