# Supplementary material for: Amino acid residue at position 188 determines the UV-sensitive bistable property of vertebrate non-visual opsin Opn5
Source: Commun Biol. 2022 Jan 18;5:63. doi: 10.1038/s42003-022-03010-x (PMC8766551; doi:10.1038/s42003-022-03010-x)
Supplement: Supplementary file 3 — Description of Additional Supplementary Files [file 42003_2022_3010_MOESM3_ESM.pdf]

Description of Additional Supplementary Files

File name: Supplementary Data 1
Description: Source data for Figure 1

File name: Supplementary Data 2
Description: Source data for Figure 2

File name: Supplementary Data 3
Description: Source data for Figure 3

File name: Supplementary Data 4
Description: Source data for Figure 4
