# Supplementary material for: Amino acid residue at position 188 determines the UV-sensitive bistable property of vertebrate non-visual opsin Opn5
Source: Commun Biol. 2022 Jan 18;5:63. doi: 10.1038/s42003-022-03010-x (PMC8766551; doi:10.1038/s42003-022-03010-x)
Supplement: Supplementary file 8 — Reporting Summary [file 42003_2022_3010_MOESM8_ESM.pdf]

nature portfolio

Corresponding author(s): Takahiro Yamashita

Last updated by author(s): Dec 10, 2021

Reporting Summary

Nature Portfolio wishes to improve the reproducibility of the work that we publish. This form provides structure for consistency and transparency in reporting. For further information on Nature Portfolio policies, see our Editorial Policies and the Editorial Policy Checklist.

Statistics

For all statistical analyses, confirm that the following items are present in the figure legend, table legend, main text, or Methods section.

n/a	Confirmed	
☒	☐	The exact sample size (*n*) for each experimental group/condition, given as a discrete number and unit of measurement
☒	☐	A statement on whether measurements were taken from distinct samples or whether the same sample was measured repeatedly
☒	☐	The statistical test(s) used AND whether they are one- or two-sided<br>*Only common tests should be described solely by name; describe more complex techniques in the Methods section.*
☒	☐	A description of all covariates tested
☒	☐	A description of any assumptions or corrections, such as tests of normality and adjustment for multiple comparisons
☒	☐	A full description of the statistical parameters including central tendency (e.g. means) or other basic estimates (e.g. regression coefficient) AND variation (e.g. standard deviation) or associated estimates of uncertainty (e.g. confidence intervals)
☒	☐	For null hypothesis testing, the test statistic (e.g. *F*, *t*, *r*) with confidence intervals, effect sizes, degrees of freedom and *P* value noted<br>*Give P values as exact values whenever suitable.*
☒	☐	For Bayesian analysis, information on the choice of priors and Markov chain Monte Carlo settings
☒	☐	For hierarchical and complex designs, identification of the appropriate level for tests and full reporting of outcomes
☒	☐	Estimates of effect sizes (e.g. Cohen's *d*, Pearson's *r*), indicating how they were calculated

Our web collection on statistics for biologists contains articles on many of the points above.

Software and code

Policy information about availability of computer code

Data collection	We used UVPC Personal Spectroscopy Software Ver. 3.92 for the collection of absorption spectra data.
Data analysis	We used Igor Pro Ver. 6 and 8 for the analysis of absorption spectra and biochemical data, BioEdit Ver. 7.0.5.3. for the sequence alignment and calculation of the sequence similarity in Fig.S1.

For manuscripts utilizing custom algorithms or software that are central to the research but not yet described in published literature, software must be made available to editors and reviewers. We strongly encourage code deposition in a community repository (e.g. GitHub). See the Nature Portfolio guidelines for submitting code & software for further information.

Data

Policy information about availability of data

All manuscripts must include a data availability statement. This statement should provide the following information, where applicable:

- Accession codes, unique identifiers, or web links for publicly available datasets
- A description of any restrictions on data availability
- For clinical datasets or third party data, please ensure that the statement adheres to our policy

All source data for the graphs in the main figures are available in the supplementary data.

Field-specific reporting

Please select the one below that is the best fit for your research. If you are not sure, read the appropriate sections before making your selection.

☒ Life sciences ☐ Behavioural & social sciences ☐ Ecological, evolutionary & environmental sciences

For a reference copy of the document with all sections, see nature.com/documents/nr-reporting-summary-flat.pdf

Life sciences study design

All studies must disclose on these points even when the disclosure is negative.

Sample size	We did not use statistical methods in the study.
Data exclusions	No data was excluded from the analysis.
Replication	Replicate experiments were successful, because similar results were obtained in each experiment.
Randomization	No randomization was needed in the study.
Blinding	Investigators were not blinded to the samples during the experiments. However, the raw data of spectral analysis were independently obtained by different investigators. Thus, investigators were blinded to the data obtained by others.

Reporting for specific materials, systems and methods

We require information from authors about some types of materials, experimental systems and methods used in many studies. Here, indicate whether each material, system or method listed is relevant to your study. If you are not sure if a list item applies to your research, read the appropriate section before selecting a response.

Materials & experimental systems

n/a	Involved in the study	
☐	☒	Antibodies
☐	☒	Eukaryotic cell lines
☒	☐	Palaeontology and archaeology
☒	☐	Animals and other organisms
☒	☐	Human research participants
☒	☐	Clinical data
☒	☐	Dual use research of concern

Methods

n/a	Involved in the study	
☒	☐	ChIP-seq
☒	☐	Flow cytometry
☒	☐	MRI-based neuroimaging

Antibodies

Antibodies used	anti-bovine rhodopsin monoclonal antibody (Rho1D4)
Validation	Anti-bovine rhodopsin monoclonal antibody (Rho1D4) was validated in previous papers (Molday & MacKenzie (1983) Biochemistry 22, 653; Molday & Molday (2014) Methods Mol. Biol. 1177, 1).

Eukaryotic cell lines

Policy information about cell lines

Cell line source(s)	HEK293S cells were kindly provided from Prof. J. Nathans (Johns Hopkins School of Medicine, Baltimore, MD).
Authentication	HEK293S cells were characterized and were authenticated as described (Nathans et al., (1989) Vision Res. 29, 907).
Mycoplasma contamination	HEK293S cells were tested negative for mycoplasma contamination.
Commonly misidentified lines (See ICLAC register)	HEK293S cell line was not listed by ICLAC.
